# Noninvasive Tools to Predict Necrotizing Enterocolitis in Infants with Congenital Heart Diseases: A Narrative Review

**DOI:** 10.3390/children11111343

**Published:** 2024-10-31

**Authors:** Laura Moschino, Silvia Guiducci, Miriam Duci, Leonardo Meggiolaro, Daniel Nardo, Luca Bonadies, Sabrina Salvadori, Giovanna Verlato, Eugenio Baraldi

**Affiliations:** 1 Department of Woman’s and Child’s Health, University of Padova, 35128 Padua, Italy; silvia.guiducci@aopd.veneto.it (S.G.); leonardo.meggiolaro@aopd.veneto.it (L.M.); luca.bonadies@aopd.veneto.it (L.B.); giovanna.verlato@aopd.veneto.it (G.V.); eugenio.baraldi@unipd.it (E.B.); 2 Neonatal Intensive Care Unit, Padova University Hospital, 35128 Padua, Italy; daniel.nardo@aopd.veneto.it (D.N.); sabrina.salvadori@aopd.veneto.it (S.S.); 3 Institute of Pediatric Research, Padova University Hospital, 35128 Padua, Italy; miriam.duci@aopd.veneto.it; 4 Pediatric Surgery, Padova University Hospital, 35128 Padua, Italy

**Keywords:** necrotizing enterocolitis, congenital heart disease, neonate, infant, multimodal monitoring

## Abstract

Background: Necrotizing enterocolitis (NEC) is the most frightening gastrointestinal emergency in newborns. Despite being primarily a disease of premature infants, neonates with congenital heart disease (CHD) are at increased risk of development. Acute and chronic hemodynamic changes in this population may lead to mesenteric circulatory insufficiency. Objectives: In this narrative review, we describe monitoring tools, alone or in multimodal use, that may help in the early recognition of patients with CHD at major risk of NEC development. Methods: We focused on vital parameters, echocardiography, Doppler flowmetry, abdominal near-infrared spectroscopy (aNIRS), and abdominal ultrasound (aUS). Results: The number of studies on this topic is small and includes a wide range of patients’ ages and types of CHD. Peripheral oxygen saturation (SpO_2_) and certain echocardiographic indices (antegrade and retrograde velocity time integral, cardiac output, etc.) do not seem to differentiate infants with further onset of NEC from those not developing it. Hypotensive events, persistent diastolic flow reversal in the descending aorta, and low mesenteric oxygen saturation (rsSO_2_) measured by aNIRS appear to occur more frequently in infants who later develop NEC. aUS may be helpful in the diagnosis of cardiac NEC, potentially showing air contrast tracked to the right atrium in the presence of pneumatosis. Conclusions: This narrative review describes the current knowledge on bedside tools for the early prediction of cardiac NEC. Future research needs to further explore the use of easy-to-learn, reproducible instruments to assist patient status and monitor patient trends.

## 1. Introduction

Necrotizing enterocolitis (NEC) is the most common gastrointestinal emergency in the neonatal intensive care unit (NICU). It is primarily a disease of premature infants, occurring in approximately 7% of extremely-low-birth-weight (ELBW) neonates. This condition carries a high burden of mortality, which approaches 30% in very-low-birth-weight (VLBW) infants and 50% in those with surgical NEC [1,2]. In this population, the main stem of disease onset is an inflammatory process of the immature bowel due to a trigger event, eventually leading to further loss of barrier integrity, bacterial invasion, and intestinal necrosis [3]. Not only premies, however, can be affected. Approximately 10–20% of NEC cases occur in term neonates, who have an almost fully mature intestine. NEC can indeed be considered the common endpoint of several pathogenetic mechanisms and pathologies (such as intrauterine growth restriction (IUGR), sepsis, asphyxia, and congenital heart disease (CHD)), eventually culminating in inflammatory coagulative necrosis of the gut [4,5].

NEC occurring in neonates with CHD, namely cardiac NEC, differs from classical preterm NEC in incidence, presentation, and pathogenetic mechanisms. Cardiac NEC was first described in 1976 in the context of ductal-dependent hypoplastic left heart syndrome (HLHS) [6]. Its overall incidence varies among studies from 3 to 5%, being approximately 10- to 100-fold higher than the incidence of NEC in the general population of term neonates admitted to NICU [7,8]. Additionally, cardiac NEC accounts for nearly 20% of normal weight (>2500 g) term NEC cases [9]. The true incidence of cardiac NEC, however, is difficult to gauge because of the rarity of the condition, the nature of published studies (mainly retrospective and single-center), and the variability of the population and cardiac lesions [10].

CHD is a known independent risk factor for NEC. A prospective collection of data from 674 Vermont Oxford Network (VON) US centers revealed that the incidence of NEC was significantly greater in VLBW infants with CHD than in those without CHD (13% vs. 9%). Additionally, the mortality of infants with CHD and NEC was greater (55%) than that of infants with either disease alone (for those with CHD but without NEC: 34%; for those with NEC but without CHD: 28%). Finally, despite a decrease in mortality of classical NEC with increasing birth weight, this decrease did not occur for mortality in CHD-associated NEC [11]. According to a retrospective review of neonates with CHD at a tertiary center (2006–2016), patients with ductal-dependent (DD) lesions presented higher rates of NEC than non-ductal-dependent patients did, but mortality rates were similar between the two groups [12].

Like classical NEC, cardiac NEC is multifactorial, but factors affecting gut perfusion play a pivotal role [13]. The relationship between heart disease and NEC in full-term infants is almost certainly circulatory, with acute and chronic hemodynamic changes being responsible. As summarized by McElhinney et al. [4], patients with critical CHD are predisposed to mesenteric circulatory insufficiency, for example because of widened pulse pressure and low diastolic pressure. This is seen preoperatively in patients with shunting at the level of the great arteries (such as in the truncus arteriosus (TA) and aortopulmonary window (APW)), as well as in those with a large patent ductus arteriosus (PDA) in a ductal-dependent circulation. Patients with this physiology have retrograde diastolic flow in the descending aorta, potentially resulting in mesenteric ischemia [14]. In contrast, other cardiac patients (i.e., those with coarctation of the aorta (CoA) and hypoplastic left heart syndrome (HLHS)) may present with circulatory collapse and acute gut ischemia upon ductal closure. Persistent or intermittent hypoperfusion, as early as delivery, as well as abnormalities in the splanchnic vasculature, have been postulated among the components of cardiac NEC. These elements are supposed to trigger endothelial inflammation and thereafter vascular permeability, in the context of a greater systemic inflammatory storm than in infants without CHD [10,15,16]. In NEC cases that develop postoperatively, instead, reperfusion injury after corrective surgical restoration of blood flow is believed to spur systemic inflammation involving reactive oxygen species (ROS) [10,17]. It has also been postulated that microcirculatory changes, such as those induced by chronic hypoxia, impair normal flow autoregulation, and this, combined with increased metabolic demands, further increases the susceptibility of CHD neonates to ischemia [10,18].

From these pathophysiological mechanisms, some noninvasive bedside tools, such as echocardiography, Doppler flowmetry of the abdominal aorta and mesenteric vessels, as well as abdominal NIRS, may help in the early recognition of CHD patients at major risk of developing NEC [19,20].

The presentation of cardiac NEC, which mostly occurs in term neonates, is usually earlier than that of classical NEC, at a median of 7–16 days (1–24 days) of life [21]. However, the onset of cardiac NEC may occur either preoperatively or postoperatively [22,23]. In a single-center experience, cardiac NEC associated with non-ductal-dependent lesions (such as TA and APW) typically occurs prior to cardiac surgery, whereas NEC associated with ductal-dependent lesions occurs more frequently postoperatively [12]. However, in another retrospective study, all non-DD patients developed NEC after congenital heart surgery [24]. Additionally, several studies have shown an association between higher doses or prolonged duration of prostaglandin (PGE_1_) infusion and the development of NEC [4,22,25].

With respect to localization, studies have shown contrasting results, with some arguing for colonic and distal ileus involvement in cardiac NEC due to blood supply interruptions in these “watershed zones” and others supporting a more ileal site of injury instead [10,26,27].

Despite the lower requirement for surgical resection of the bowel, neonates with cardiac NEC have an increased mortality risk for all causes, compared with classical NEC infants, which can reach 39% and 57% in noncyanotic and cyanotic CHDs, respectively [28].

Instrumental tools, such as NIRS or echocardiography, need further exploration in clinical research, as they may become extremely useful in the prediction and anticipation of NEC in this high-risk population. This narrative review focuses on the current knowledge of predictive tools for the early recognition of NEC in neonates and infants with CHD.

Table 1 shows the features of classical vs. cardiac NEC.

## 2. Materials and Methods

For the purpose of this narrative review, we conducted a systematic database search (PubMed, MEDLINE Ovid, Scopus) from inception to August 2023. Three reviewers (L.M., S.G., M.D.) independently performed the search using the following terms: “neonate” OR “neonates” OR “newborn” OR “newborns” OR “infant” OR “infants” AND “congenital heart disease” OR “heart disease” OR “CHD” AND “necrotizing enterocolitis” OR “NEC”. Additional articles were identified by a manual search of the cited references. Among the emergent studies, those focusing on predictive tools, used in combination or alone, to identify neonates (<28 days of age, any gestational age) or infants (<1 year of age) with CHDs at risk of NEC were selected. These tools included: vital parameters (SpO_2_, heart rate HR, blood pressure BP, and pulsatility index PI), echocardiographic measures (such as cardiac output CO, antegrade and retrograde VTI, etc.), Doppler flowmetry of the abdominal descending aorta and/or its trunks, aNIRS with regional SpO_2_, and aUS.

**Figure 1:**
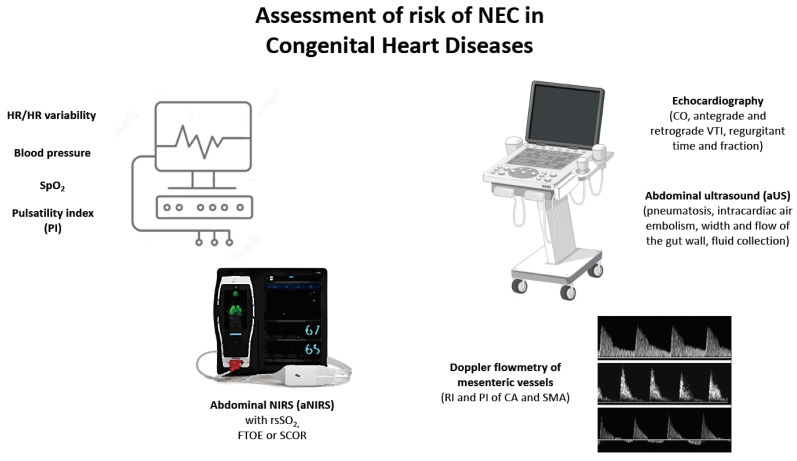
Potential bedside, easy-to-perform, point-of-care tools that might be used to predict the risk of NEC onset in patients with CHDs and that have been explored in some studies enrolling neonates and infants with variable findings. Image by Freepik and Biorender. Abbreviations: aNIRS = abdominal near-infrared spectroscopy; aUS = abdominal ultrasound; CA = celiac artery; CO = cardiac output; FTOE = fraction of oxygen extraction; HR = heart rate; PI = pulsatility index; RI = resistive index; SCOR = splanchnic to cerebral oxygenation ratio; SMA = superior mesenteric artery; VTI = velocity-to-time integral.

Titles and abstracts were screened for relevance and duplications by the three reviewers independently, with disagreements resolved by discussion with a fourth reviewer. Randomized control trials, when present, as well as retrospective and prospective cohort studies, were considered. Given the scarcity of RCTs and longitudinal studies on the topic, case series and reports were also included for quality assessment. Unpublished studies (e.g., conference abstracts) and animal studies were excluded. Abstracts with full texts in a language other than English were excluded. Cardiac NEC was defined according to Bell’s stage in patients with CHDs or as per the author definition reported in the study. We excluded articles considering only classical NEC (i.e., NEC related to prematurity) and articles including only patients having a PDA as cardiac lesion. Figure 1 summarizes the tools that have been considered potentially useful for this purpose. More details on the database search and on the selection of studies can be found in the Supplementary Materials.

## 3. Results

In the determination of the risk of NEC in CHD patients, the anatomical and pathophysiological classification of CHD must be considered.

The first group of CHD patients at high risk of NEC includes infants with increased pulmonary blood flow due to unobstructed mixing lesions with left-to-right shunt, such as truncus arteriosus (TA), aortopulmonary window (APW), large ventricular septal defects (VSDs), atrioventricular septal defects (AVSD), and double-outlet right ventricle (DORV) with VSD physiology. Under these conditions, the initial nonobligate mixing may progressively shift toward a large left-to-right shunt as pulmonary vascular resistance (PVR) decreases in the first 3–4 weeks of life. As a consequence, pulmonary overcirculation and systemic steal due to a high pulmonary-to-systemic flow ratio (Qp/Qs) may occur, increasing the risk of systemic hypoperfusion. In the study by McElhinney et al. [4], TA and APW represented 14% of all cardiac NEC cases. A prospective analysis of VON US centers, instead, reported a higher incidence of NEC in infants with atrioventricular canals [11].

A second group of CHDs with a high risk of NEC is characterized by ductal-dependent (DD) lesions, either due to pulmonary or systemic obstructive lesions or due to parallel pulmonary and systemic circulation, such as in complete transposition of the great arteries (dTGA). Tricuspid/pulmonary atresia, hypoplastic right ventricle, severe tetralogy of Fallot (TOF), and severe Ebstein’s anomaly have DD pulmonary circulation, possibly determining low peripheral oxygen saturation. Severe mitral stenosis/regurgitation, severe/critical aortic stenosis, hypoplastic left ventricle (HLHS), aortic coartation (CoA), and aortic arch interruption (AAI) instead have DD systemic circulation with possible systemic hypoperfusion before prostaglandin infusion is started and surgical correction occurs. In particular, some of these lesions (CoA, MR, or MS) may be undetected in the prenatal period; thus, they may present with acute cardiogenic shock and multiorgan failure, comprising NEC.

Patients with aortic arch obstruction accounted for 57% of all NEC cases in the cohort by McElhinney et al. [4]. Similarly, patients with a functional univentricular anomaly, particularly those with HLHS, represent a high percentage of all cardiac NEC cases, accounting for approximately 38–62% and 24–48% of the total cases, respectively, according to previous studies [4,22]. Among infants with HLHS, the incidence of NEC is estimated to be as high as 7.6%, whereas it is approximately 2.1% in patients with other cardiac lesions [4]. HLHS puts these neonates at risk of NEC both before and after stage I Norwood palliation. Even after stage I, the modified Blalock–Taussig shunt (mBTS) may limit the aortic and mesenteric flow due to diastolic runoff from the systemic to pulmonary circulation, similar to what is observed in preterm infants with a hemodynamically significant patent ductus arteriosus (HsPDA) [31]. In these patients, the reported incidence of NEC has been 7–15% [32,33]. The hybrid procedure, consisting of bilateral banding of the pulmonary arteries and stenting of the DA, despite being less invasive and not requiring cardiopulmonary bypass (CPB) and deep hypothermic circulatory arrest (DHCA), has not been proven to reduce the incidence of NEC, which was 11% in one study of 73 patients with complex CHDs [34,35].

Finally, as already mentioned, NEC can be a complication of acute cardiogenic shock in dilated/restrictive cardiomyopathy, myocarditis, or post heart surgery, in which a low cardiac output state can determine gut hypoperfusion and subsequent necrosis [4].

**Figure 2:**
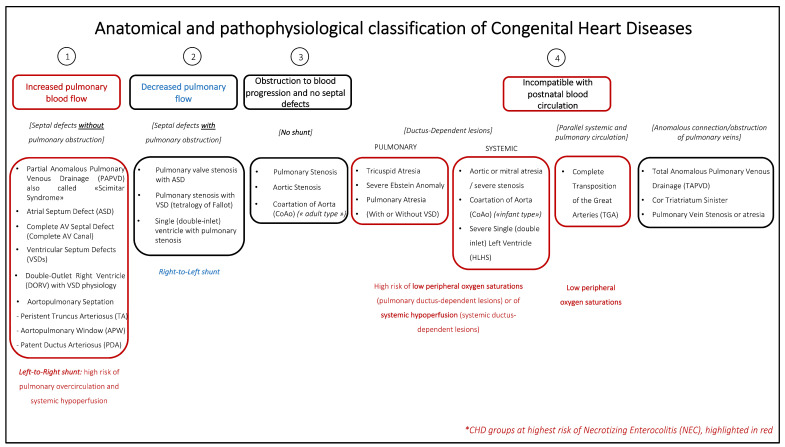
Anatomical and pathophysiological classification of CHDs. Cardiac anomalies are divided as follows: (1) CHD with increased pulmonary blood flow (septal defects without pulmonary obstruction and with L-t—R shunt; (2) CHD with decreased pulmonary flow (septal defects with pulmonary obstruction and with R-to-L shunt); (3) CHD with obstruction to blood progression and no septal defects (no shunt); (4) CHD so severe as to be incompatible with postnatal blood circulation (ductus-dependent CHD, parallel systemic and pulmonary circulations, anomalous connection/obstruction of the pulmonary veins). Adapted from Thiene G, Frescura C. Cardiovasc Pathol 2010 [36].

Figure 2 depicts a classification of CHDs according to their anatomy and pathophysiology, with those with a higher risk of NEC in red.

### 3.1. Vital Parameter Monitoring

Some vital parameters may be indirect indicators of intestinal hypoperfusion. Recent studies have demonstrated that the analysis of continuous vital sign trends, instead of punctual measurements, can predict different neonatal morbidities, including NEC and mortality [37].

#### 3.1.1. Heart Rate (HR), HR Variability, and HR Characteristics (HRCs)

In newborns born at <1500 g or preterm, but without CHD, abnormal heart rate characteristics (HRCs) such as decreased variability and transient decelerations can predict subsequent diagnoses of medical or surgical NEC [38]. In another small study, a decreased high-frequency (HF) component of the heart rate variability (HRV) (a measure of vagal efferent tonic cholinergic activity) in the first week after birth was associated with a later diagnosis of NEC [39]. Another study revealed that a cross correlation between HR and oxygen saturation (SpO_2_) was associated with apnea and adverse events, including NEC [40].

#### 3.1.2. Blood Pressure (BP)

Systemic hypotension is frequently described in infants with CHD and was associated with a higher risk of NEC in three studies [4,41,42]. Leung et al. showed that hypotension associated with prostaglandin infusion was a risk factor for NEC in patients with CHD, postulating that the drug may lead to these symptoms due to the consequent diving reflex and intestinal ischemia [41]. In contrast, Hebra et al. reported that hypotension or cardiac arrest occurred within 48 h of the onset of mesenteric ischemia in 25 patients (80%) with HLHS [42].

#### 3.1.3. Peripheral Oxygen Saturation (SpO_2_)

With respect to SpO_2_, only two studies, both retrospective, case-control studies, have investigated its fluctuation in CHD infants; however, a definitive conclusion has not been reached. Van der Heide et al. [20] compared neonates with CHD and NEC with neonates with similar CHD without NEC. They collected diastolic and systolic blood pressure and postductal SpO_2_ data during the first 48 h of hospitalization and before the onset of NEC. The authors were not able to demonstrate a statistically significant difference in these parameters between the two groups. Choi et al. [43] compared infants with DD CHD with or without NEC and analyzed SpO_2_ 12 h before its onset and 12 h before the end of PGE_1_ infusion. In this study, a significant difference was not found between the two groups. Both studies had several limitations that could hinder consistent results, including the small sample size and the low incidence of NEC, the expression of SpO_2_ being measured as the average of all SpO_2_ values over time (hiding possible episodes of hypoperfusion and hypoxia), and finally, measurement being performed at the extremity of the body (therefore not specific to gut perfusion).

#### 3.1.4. Pulsatility Index (PI)

Another measure that can be used to monitor neonatal hemodynamic status is the perfusion index (PI), which is the ratio between the pulsatile light absorption of arterial pulsatile vessels (alternating current, AC) and the continuous light absorption from nonpulsatile vessels (direct current, DC) from the photoplethysmogram signal. This tool has been used as a noninvasive surrogate of peripheral perfusion in preterm neonates, and it has been explored as a predictor of NEC in this population [44]. Nevertheless, there are no data concerning its application for the same purpose in neonates with CHDs.

Overall, studies evaluating vital sign monitoring for the prediction of NEC in CHD patients are scarce in number and have very small sample sizes, with inconsistent results. Despite being routinely used in everyday practice, these indirect measures of peripheral perfusion may vary according to temperature variation, the administration of vasoactive drugs, the tone of the sympathetic nervous system (pain, anxiety), changes in stroke volume, compression of the extremities, and positional changes (prone/supine). These limitations highlight the need for research on other tools that can specifically investigate intestinal perfusion, such as ultrasound of the mesenteric system and abdominal/splanchnic NIRS.

### 3.2. Doppler Ultrasonography

Doppler ultrasound of the abdominal descending aorta (AAo), celiac artery (CA), and superior mesenteric artery (SMA) allows direct assessment of gut perfusion and could therefore be useful in the prediction of NEC. For this purpose, several parameters have been investigated, including peak systolic velocity (PSV), end-diastolic velocity (EDV), time-averaged mean velocity (TAMV), differential velocity (DV, calculated as PSV-EDV), systolic velocity ratio (CA/SMA PSV), resistive index (RI, calculated as (PSV-EDV)/PSV) and the pulsatility index (PI, calculated as (PSV-EDV)/mean velocity). All the parameters depend on a good incident angle of the probe US waves with respect to the flow direction to minimize the error of the velocity measurement. Additionally, some of these parameters, such as PSV and EDV, and their derived RIs, PIs, and DVs, are highly influenced by vasoconstriction.

Both in animal models and in preterm neonates, abnormal splanchnic circulation has been observed in those at risk of or developing NEC [45,46,47,48], but sometimes with opposite findings. Indeed, in the first week of life, a lower [45,48] or higher [46] PSV, a lower EDV [45,48], and an increased DV [46] have been observed in infants who subsequently develop NEC. Instead, the ratio between CA-PSV and SMA-PSV (CA/SMA PSV), an indicator of downstream vascular resistance in the vascular bed of the SMA, appears to be greater in preterm infants at risk of NEC than in those without [45,48], demonstrating a pattern of hypoperfusion before the onset of the disease, which is different in the two districts supplied by these vessels.

However, some studies have reported an increase in the blood flow velocity of the SMA at disease onset [44]. This result may be explained by a gradual increase in the SMA-PSV after NEC development as a consequence of vasodilatation secondary to post ischemic hyperaemia [45,47,49].

Patent ductus arteriosus (PDA) is a known risk factor for NEC. Patients with a significant PDA have absent or reverse diastolic flow in the SMA but not in the CA, indicating compromised perfusion secondary to diastolic steal by pulmonary circulation [50]. Additionally, the effect on the blood flow velocity of the SMA seems to be greater in the postprandial period in ELBW infants with a large PDA [51].

The majority of studies assessing Doppler flow velocities in infants with CHD have been conducted in patients with HLHS undergoing first-stage palliative procedures, thus including an older population beyond the neonatal period. Harrison et al. [52] measured the Doppler parameters of the SMA 24–48 h before and after the modified Norwood procedure with a Blalock–Taussig (BT) shunt and reported that the RIs of the SMA ((PSV-EDV)/PSV) were >25% greater than normal (0.85–1.0) in these subjects, confirming reduced intestinal perfusion in neonates with this cardiac lesion. These authors did not find an improvement in the diastolic blood flow of the SMA after surgery; nevertheless, no patients developed NEC. In the prospective study by Cozzi et al., Doppler indices of perfusion (effective and antegrade velocity-time integral (VTI)) in the CA and in the SMA seemed to be ameliorated after the hybrid procedure, instead [53]. However, there appeared to be preferential flow to the CA versus the SMA post procedure, indicating that the region perfused by the SMA (mainly the duodenum, jejunum, last part of the small intestine, beginning of the colon, transverse colon, and cecum) may be at greatest risk of NEC. This study was underpowered to determine an association between NEC risk and mesenteric flow characteristics and did not evaluate pre- and postprandial changes in velocities. Some authors have suggested that both the hybrid procedure and the Norwood stage with a systemic-to-pulmonary shunt result in a persistent absent or retrograde flow in the SMA during diastole [52,54] compared with infants undergoing the Norwood stage with an RV-to-PA conduit [55]. Johnson et al. demonstrated a lower RI of the CA in those treated with an RV-to-PA conduit compared to those treated with a BT shunt, but there were no differences in gastrointestinal outcomes, chief of which was NEC [31]. When these three options of surgical treatment were compared in patients with aortic atresia after stage II palliation, no echocardiographic parameters predicted mortality or morbidity measures (renal and liver insufficiency, but not NEC) [56]. Interestingly, despite the basal and postprandial absent or retrograde diastolic flow in the SMA after a systemic-to-pulmonary shunt, infants undergoing the Norwood stage show a decrease in the basal and postprandial resistance of the splanchnic circulation, probably as an adaptive mechanism to counteract diastolic steal [54].

Other studies evaluating Doppler parameters in CHD neonates with/without NEC have reported contrasting results.

A retrospective chart review conducted in 2012 did not find any Doppler indices (e.g., antegrade VTI, retrograde VTI, effective VTI, VTI regurgitant fraction, VTI retrograde/VTI antegrade ratio, calculated cardiac output (CO), peak antegrade velocity through the ductal stent, retrograde/antegrade time ratio, and percent regurgitant time) sensitive enough to determine the risk of NEC Bell stage ≥II in infants with complex single-ventricle anatomy undergoing stage I palliation [57].

Similarly, van der Heide et al. did not find a significant difference in diastolic backflow in the DAo by echocardiography at the time of admission between near-term neonates with CHD who developed NEC and those who did not [20]. Carlo et al. [14] instead reported that a persistent retrograde diastolic flow in the abdominal aorta was associated with a greater risk of NEC in term neonates with CHDs (47% vs. 15%; variable types of lesions, including DORV, TGA, CoAo, TA, TOF, Ebstein’s, HLHS, AoS), even after correction for multiple risk factors (such as gestational age and anatomic lesions). Patients who developed NEC were fed significantly earlier than the control subjects in this study, but this factor was not associated with NEC development. These results were confirmed by cardiac magnetic resonance (CMR), but not echocardiography, by Papneja et al. [58] in a specific population of neonates with small left-sided structures. By CMR, a DAo flow of 0.91 L/min/m^2^ identified patients who experienced feeding intolerance or NEC with a sensitivity of 61% and a specificity of 76%.

With respect to the pulsatility index (PI) ((PSV-EDV)/mean velocity), findings have not been uniform, possibly due to the extreme heterogeneity of studies’ populations and timings of evaluations. Miller et al. [19] reported a lower abdominal aorta PI before and after the first palliative intervention in patients with HLHS who developed NEC during a mean follow-up of 3.8 years. Pham et al. [59], instead, reported a higher PI as a risk factor for NEC in neonates undergoing cardiac surgical repair within the first month of life.

Unfortunately, the small number of studies, the retrospective design of most of them, and the small sample sizes hamper the ability to draw firm conclusions on the use of Doppler flowmetry to predict NEC in patients with CHDs. Nevertheless, some indices of Doppler flowmetry could be promising, including the PI, RI, and diastolic flow % detected at abdominal DAo, SMA, and CA.

### 3.3. Abdominal Near-Infrared Spectroscopy (aNIRS)

Near-infrared spectroscopy (NIRS) is a noninvasive bedside monitoring tool for regional tissue oxygenation that uses static sensor placement on the abdominal surface. NIRS uses near-infrared light to measure the amount of oxygenated and deoxygenated hemoglobin to assess the balance between the oxygen request and supply in different tissues, such as the brain, gut, and kidneys [60,61]. Given the possible role of impaired intestinal perfusion as a mechanism of NEC development, especially in infants with CHD, NIRS may be a promising tool for the early detection of hypo-perfused states leading to a greater risk of NEC before or after CHD repair [62]. To date, only small studies have trialed the use of splanchnic NIRS (sNIRS) as a surrogate marker of impaired intestinal oxygenation and assessed the relationship between regional splanchnic oximetry (rsSO_2_) values and the development of NEC [63,64,65,66]. Detecting real splanchnic oxygenation with aNIRS can be challenging because of the possible variability of recordings and the low reproducibility [67]. Unlike the brain and kidneys, which are fixed within the skull and retroperitoneum, the gut is extremely mobile in the abdominal cavity, making it difficult to detect the precise regional oxygenation of bowel loops through external sensors. The measurement may be further complicated by the gas–fluid surface, the variability of sensor placement on the abdomen, and the potential effect of feeding on splanchnic perfusion [64,68]. Additionally, enteral saturation has the limitation of the impossibility of full contact of the sensor with the underlying tissues of the gastrointestinal tract, in contrast to the possibilities of applying NIRS to detect kidney and brain saturation.

Despite these limitations, which highlight the need for further research on the topic, a unique recent large cohort study on preterm infants (n = 220, <32 w and/or <1500 g BW) revealed that gestational age (GA), postnatal age, and small-for-gestational age (SGA) status significantly affect regional splanchnic oxygen saturation [63]. Van der Heide et al. provided a model so that reference values for infant regional splanchnic oxygen saturation can be computed with their formula on the basis of those factors. Specifically, they demonstrated that the nadir of the rsSO_2_ was on day 4 (38.7% ± 16.6), after which the mean rsSO_2_ increased to 44.2% (±16.6) on day 7. There are no similar studies performed in term newborns or in newborns with CHDs. However, some evidence has shown the utility of monitoring trend values, as well as of the derived measures of splanchnic-cerebral oxygenation ratio (SCOR) and fraction of oxygen extraction (FTOE), in guiding the diagnosis and detection of pathological conditions [69,70].

The current literature evaluating the relationship between rsSO_2_ and risk of NEC onset provides contrasting results. Some studies reported that a persistently low rsSO_2_ value with low variability in the first two postnatal weeks was a risk factor for NEC development in premature infants [64,65]. Schat et al., instead, showed that sNIRS monitoring might be helpful in distinguishing complicated NEC from uncomplicated cases but not in differentiating patients with or without NEC [66]. Similarly, a recent prospective study demonstrated that NIRS monitoring was unable to identify NEC patients or those who developed a more severe disease at the time of suspicious gastrointestinal symptoms [71].

NIRS monitoring in the context of CHD repair has been applied both intraoperatively and postoperatively to assess tissue perfusion [72,73]. However, only a few studies have focused on rsSO_2_ monitoring. The first report of a single case supporting the correlation between mesenteric desaturation, as detected by aNIRS, and the development of NEC was published in 2007. In this report, the authors demonstrated that NIRS monitoring can be used to early detect regional tissue hypoxia after cardiac surgical repair [74]. After this case report, other studies evaluated the relationship between rsSO_2_ plus markers of low cardiac output after surgery and early postoperative outcomes [75], with both positive [76] and negative [77] correlations, but the association with NEC was not explored.

A more recent prospective study was the first to assess splanchnic oximetry in a cohort of neonates with complex CHD and its relationship with NEC development [78]. The enrolled neonates had undergone either biventricular repair or single ventricle (SV) palliation, with none of the former group developing NEC compared with 32% of the latter. The authors’ findings showed that, compared with the patients who had suspected or no NEC (no NEC or Bell’s stage I), the subjects with proven NEC had a significantly lower average rsSO_2_ (32.6% vs. 47.0%), more time spent with rsSO_2_ <30% (48.8% vs. 6.7%) at one-fourth-volume feeds, and more time with SpO_2_-rsSO_2_ exceeding 50% (33.3% vs. 0%) before feeds were initiated. These data, as suggested by the authors, demonstrate that splanchnic NIRS may be a useful tool for assessing the risk of NEC, especially in patients with an SV physiology.

Overall, these studies indicate that sNIRS is still underexplored in neonates and infants with CHD before or after surgery. Nevertheless, evidence from studies conducted mainly in preterm infants shows its potential utility as a surrogate of gut perfusion, as it can detect hypoxia after CHD repair, may correlate with certain markers of low CO, and may be altered in patients with SV physiology who develop NEC. Larger randomized trials are warranted to validate the use of this technique and to standardize its application.

### 3.4. Abdominal Ultrasound (aUS)

Abdominal ultrasound (aUS) is progressively overcoming the role of abdominal radiography (aXR) in diagnosing NEC in newborns. Indeed, in 2018, the NEC group of the International Neonatal Consortium recommended the use of aUS to detect pneumatosis and/or portal air in preterm NEC as part of the ‘two out of three’ model [79]. Furthermore, the international evidence-based guidelines of point of care ultrasound (POCUS) of the ESPNIC suggest its use to detect signs of NEC, assess bowel peristalsis, and detect free abdominal fluid [80]. This noninvasive bedside technique may also be applied in CHD patients.

A recent study conducted by the Boston Children’s Hospital group revealed that CHD patients with NEC were less likely to have US findings of pneumatosis and of decreased mural flow than non-CHD patients with NEC were [81]. No other significant differences in the rates of pneumatosis on aXR or any other radiological or aUS findings (portal venous gas, echogenic or hyperemic bowel, pneumoperitoneum and fluid collection) emerged.

Regarding uncommon aUS signs, an interesting report published in 2012 described the case of a 5-day-old term neonate with Down syndrome affected by a complete atrioventricular septal defect, in whom a stream of air contrast was tracked from the hepatic veins. This was thereafter proven to be present because of pneumatosis in the course of NEC [82]. Air bubbles in the right atrium were considered suspicious signs of NEC even by Müller et al., who described the case of a 5-week-old boy with pulmonary atresia and intact ventricular septum (PA-IVS) with gastric pneumatosis due to cardiogenic NEC. Intracardiac air was found on echocardiogram and speculated to have moved from the gastric wall through the connecting veins to the right atrium [83]. A similar finding of systemic air embolism was recognized in a 6-day-old neonate with complex CHD who developed fulminant NEC [84].

In conclusion, it must be emphasized that aUS signs of NEC are late findings and could be helpful in the differential diagnosis rather than in the risk prediction of this condition. Pneumatosis and decreased mural flow may be less common in this population, where intracardiac air may be a characteristic of pneumatosis. Although aUS is more sensitive for some signs, it is more time intensive and needs advanced training to be reliable.

Table 2 summarizes the aforementioned noninvasive tools for the prediction of NEC in CHD infants that emerged from the selected studies.

## 4. Discussion

Among the 10% of NEC cases that occur in term neonates, CHD is a known and important risk factor. Prematurity and overfeeding may put the population of cardiac patients in further danger. In fact, the incidence of NEC is significantly greater in VLBW infants with CHD than in those without CHD (13% vs. 9%), and the mortality of preterm infants with CHD and NEC is greater than that of preterm neonates with NEC alone (43–55% vs. 30%).

Compared with preterm NEC infants, affected infants with PDA or CHD and NEC present greater levels of macroscopic intestinal necrosis and intraoperative invading bacteria [85].

Certain types of critical CHD that are more commonly associated with NEC due to poor systemic blood flow include lesions with pulmonary overcirculation and systemic steal (TA, APW), left sided obstructive heart lesions with impaired systemic blood delivery (CoA, AAI, MS/MA), and, most importantly, HLHS both before and after palliation with a shunt.

The majority of studies evaluated in this narrative review have considered neonates < 30 days of age, although some studies have also included patients at older ages (e.g., after stage II HLHS or Fontan). Additionally, one study compared neonates with NEC with or without CHDs but included preterm infants, thus a different pathophysiological type of NEC was used as well [81]. Vital parameters alone may not recognize infants at risk of further NEC development. Low diastolic BP, hypotensive episodes, and prolonged PGE_1_ infusion therapy, instead, could be associated with a greater risk of the disease.

Compared with infants with CHD without NEC, those with CHD and NEC may have persistent abnormal flow patterns as indicated by diastolic flow reversal in the abdominal aorta, detected both with echocardiography and with CMR [14,58]. Data on the pulsatile flow and its resistance (PI, RI) in minor vessels, such as the SMA or the CA, have been more variable and hinder the ability to draw firm conclusions. In particular, the utility of the Doppler flowmetry of these vessels has been predominantly explored in patients with HLHS at different stages of palliative correction, and some of them were underpowered to detect association with NEC onset. Therefore, further studies need to address the utility of these parameters to monitor CHD patients.

aNIRS has been applied both intraoperatively and postoperatively to assess tissue perfusion [72,73]; however, very few studies have focused on rsSO_2_ monitoring, and the majority of these have been conducted in neonates after single-ventricle palliation, where subjects developing NEC showed a lower average splanchnic rsSO_2_, more time with rsSO_2_ less than 30% at one-fourth-volume feeds, and more time with SpO_2_-rsSO_2_ exceeding 50% before feeds initiation [78]. The use of SCOR and FTOE should be implemented in these subjects, as reference values of rsSO_2_ according to cardiac lesion may be difficult to extrapolate.

Finally, similar pathogenic findings can be found at aUS and aXR in infants with NEC, with or without CHD. Some case reports specifically on CHD neonates have described the presence of intracardiac air as a surrogate of intestinal pneumatosis in these patients.

Well-designed, potentially multicentered, studies should be conducted to shed more light on this topic. Standardization of care, with strict feeding protocols and continuous monitoring of vital parameters and measures of organ perfusion, may help improve the outcomes in this fragile population.

## 5. Conclusions

This narrative review describes the current knowledge of bedside predictive tools for the early recognition of cardiac NEC. Additionally, the field is still underexplored, and multicenter, well-designed randomized controlled trials may be useful in selecting those tools that offer greater benefit in monitoring CHD patients. Point-of-care, bedside, easy-to-learn, reproducible tools, alone or integrated, may help in the management and the identification of therapeutic approaches for this fragile population (e.g., through special preoperative and postoperative feeding protocols, use of blood biomarkers, and echocardiographic measures of low cardiac perfusion).

## Figures and Tables

**Table 1:** Characteristics of cardiac NEC compared with those of classical NEC [13,22,25,29,30].

	Classical NEC	Cardiac NEC
**Population**	Preterm	Preterm, term
**Onset**	Later presentation (4–5 weeks of life)	Earlier presentation (1–3 weeks of life)
**Risk factors**	-Low BW and GA -Feeding -Dysbiosis -Asphyxia	-Low BW and GA -Systemic ductal-dependent lesions (CoAo, AAI) -Low cardiac output state/shock -High dose (>0.05 μg/kg/minute) or long duration of PGE_1_ -Specific CHD pathologies (HLHS, TA, APW, AVSD) -Trisomy 21
**Localization**	Ileus, colon, pan-NEC	Colon, distal ileus, pan-NEC

**Table 2:** Summary of potential noninvasive predictive tools oforNEC in neonates/infants with CHD.

Potential Predictive Tool	Article Included Population	Study Type and Population	Main Findings
**SpO_2_**	*Van der Heide* et al. *BMC Pediatr 2020* [20] *Neonates*	Retrospective case-control study on neonates > 35 GW with CHD; 16 CHD patients with NEC (GA 38.6, BW 2937 g) vs. 16 matched CHD controls without NEC (GA 39.1, BW 3323 g) (TGA, ToF, CoAo, IAA, PVS)	No difference in median diastolic and systolic blood pressure and post-ductal SpO_2_ during the first 48 h of hospitalization and before the onset of NEC between cases and controls
	*Choi* et al. *BMC Pediatr 2022* [43] *Neonates*	Retrospective case-control study; 355 full-term infants (≥37 GW) with DD-CHD receiving PGE_1_ within 7 days from birth (10 CHD-NEC, GA 37.9, BW 2783.5 g; 325 no-NEC, GA 39.1, BW 3170.9)	Clinical risk factors: GA < 38 weeks, BW < 2500 g, need for MV, parenteral nutrition, functional single ventricle; no difference in SpO_2_ in the 12 h before the onset of NEC and in the 12 h before the end of PGE_1_ infusion between infants with or without NEC
**Blood pressure or episodes of hypotension**	*Leung* et al. *J Pediatr 1988* [41] *Infants*	Prospective observational study; 133 infants with symptomatic CHD (7 with NEC) (GA, BW, time of evaluation not found)	**PGE_1_ infusion (*p* = 0.02)**, and apnea (*p* < 0.01) and hypotensive episodes (*p* < 0.001) **induced by PGE_1_ infusion as risk factors for NEC**
	*Hebra* et al. *J Pediatr Surg 1993* [42] *Infants*	Retrospective case-control study; 387 patients undergoing Norwood stage I for HLHS (31 with mesenteric ischemia, mean age at evaluation 17.5 weeks, 87% full-term, mean BW 3 kg)	Significant hypotension or low perfusion state documented within 48 h prior to the onset of mesenteric ischemia in 80% of patients (mortality of 97%)
	*McElhinney* et al. *Pediatrics 2000* [4] *Neonates*	Retrospective case-control study; original cohort: 643 patients < 1 month of age with structural, electrical, and/or myocardial/metabolic heart disease; of these, 21 patients with NEC (GA 36.7, BW 3 kg, age at admission 5.1 d) and 70 matched controls (GA 38.1, BW 2.8 kg)	Clinical risk factors: prematurity (<36 weeks), and GA; **episodes of low cardiac output** (based on laboratory criteria: metabolic acidosis with arterial pH < 7.2, serum creatinine > 1.2 mg/dL; and serum aspartate aminotransferase and serum alanine aminotransferase > 300 U/L) **or clinical shock** (not meeting these criteria) (*p* = 0.005, OR 6.5), or **highest PGE dose > 0.05 mcg/kg/min** (*p* = 0.04, OR 3.9) correlated with the development of NEC;
**Heart rate and heart rate variability**	*No studies including neonates/infants with CHD*		
**Perfusion index**	*No studies including neonates/infants with CHD*		
**Doppler indices of abdominal and mesenteric vessels **	*Johnson* et al. *Pediatr Cardiol 2011* [31] *Neonates*	Observational study of 44 patients from 5 centers belonging to the Pediatric Heart Network Single Ventricle Reconstruction Trial (MBTS: n = 19, GA 38, BW 3.15 kg, age at surgery 4 d; RVPAS n = 25, GA 38, BW 3.1, age at surgery 6)	**Median RI of the CA higher in the MBTS group than in the RV-to-PAS group** (1 vs. 0.82, *p* = 0.02); no difference in NEC or feeding intolerance episodes
	*Cozzi* et al. *Congenit Heart Dis. 2013* [57] *Neonates*	Retrospective chart review of 69 patients undergoing the hybrid procedure for single-ventricle palliation (8 with NEC Bell’s stage > or =2, GA 35.5, BW 2.6 kg vs. GA 38, BW 3.1 kg)	After the hybrid procedure, **no differences between the NEC and No NEC groups for the echocardiographic indices** (antegrade VTI, retrograde VTI, effective VTI, VTI regurgitant fraction, VTI retrograde/VTI antegrade ratio, CO, peak antegrade velocity through PDA stent, retrograde/antegrade time ratio, % regurgitant time)
	*Carlo* et al. *Pediatrics 2007* [14] *Neonates*	Retrospective case-control study of CHD patients (variable diagnoses), 18 with NEC Bell’s stage > or =2 (GA 37.4, BW 2952 g, age at onset 5 d (1–24)) and 20 controls (GA 37.8, BW 3103 g)	Persistent diastolic flow reversal in the descending aorta associated with increased likelihood of NEC in term neonates with CHD (47% vs. 15%, OR 5.04)
	*Papneja* et al. *In J Cardiovasc Imaging 2021* [58] *Neonates*	Retrospective study of 51 neonates with small left-sided structures (including borderline left ventricle and HLHS), with preoperative cardiac MRI and abdominal Doppler US, of which 13 with feeding intolerance and 2 with NEC (composite outcome) (45 term neonates, 5 born 35–37 weeks, 1 born 33 + 5 weeks; mean age at evaluation 4.6 d)	**Significant lower mean DAo flow by MRI in patients with the composite outcome** vs. controls (0.89 L/min/m^2^ vs. 1.23 L/min/m^2^, *p* = 0.007); a DAo mean flow of 0.91 L/min/m^2^ identified patients with the composite outcome with Se 61% and Sp 76%; no correlation between DAo flow by MRI and AUS; no association between DAo flow by AUS (PI, PSV or MV) and the composite outcome
	*Van der Heide* et al. *BMC Pediatr 2020* [20] *Neonates*	Retrospective case-control study of 32 neonates with CHD; 16 CHD patients with NEC (GA 38.6, BW 2937 g) vs. 16 matched CHD controls without NEC (GA 39.1, BW 3323 g) (TGA, ToF, CoAo, IAA, PVS)	No difference in diastolic backflow in the DAo between the two groups
	*Miller* et al. *Pediatr Cardiol 2014* [19] *Neonates/Infants*	Retrospective cohort study of 61 neonates/infants undergoing stage I palliation for HLHS or other right single-ventricle anomaly at any time frame until Fontan correction; 11 with NEC (BW 3126 g) and 50 controls (BW 3130 g) (mean follow-up 3.8 y); NEC occurred pre-operative (n = 1), prior to stage I discharge (n = 3), between stage I and stage II (n = 5), post-stage II (n = 1), and post-Fontan (n = 1)	No difference in gender, BW, baseline anatomy, surgical procedure, bypass time or cross-clamp time; at both pre-operative and routine post-operative stage I echo, **lower abdominal aorta PI in the NEC group** (any time of onset) compared to the no NEC group (pre-operative: 3.38 vs. 3.89 with *p* < 0.05; post-operative: 2.21 vs. 3.05 with *p* = 0.01); no difference in RV function nor GLS
	*Pham* et al. *Pediatr Cardiol 2022* [59] *Neonates*	Retrospective single-institution case-control study of patients with CHD (<30 days of age, variable diagnoses) undergoing surgical repair within the first month of life, 30 developing NEC (GA 38.4, BW 3.3 kg) and 50 controls (GA 38.8, BW 3.2 kg)	From Doppler of the DAo, **median log PI of the NEC group higher than the lowest control PI** (0.68 vs. 0.48, *p* = 0.03), and **lower than the highest control PI** (0.61 vs. 0.98, *p* = 0.05); higher median log modified PI (MODPI) of the NEC group vs. that of the control group (3.9 vs. 3.1, *p* = 0.01, where negative values due to retrograde flow are substituted with zero)
**Abdominal (splanchnic) NIRS**	*Stapleton* et al. *Tex Heart Inst J 2007* [74] *Neonate*	Single case report of infant with PA and IVS and MAPCAs developing NEC at 4 weeks of life (term neonate, 2.4 kg; 120 cc/kg/day oral feeds at NEC diagnosis)	**Low mesenteric SO_2_ compared to cerebral SO_2_** measured by splanchnic NIRS 48 h after the diagnosis of NEC (24.5% vs. 53.4%)
	*DeWitt* et al. *Pediatr Cardiol 2014* [78]	Prospective study on 64 neonates undergoing BV repair or SV palliation for CHD, monitored postoperatively by rsSO_2_ before and during initiation of enteral feeds; 11 developing NEC, 23 not developing NEC (Weight at surgery 3.3 vs. 3.3 kg)	- Different incidence of NEC (BV repair 0%, SV palliation 32%), similar clinical parameters in the SV group apart for CPB time longer in those developing NEC (*p* = 0.03) - Significantly lower rsSO_2_ before and during initiation of enteral feedings in neonates after SV palliation vs. neonates after BV repair, but not after correction for SpO_2_ - **In subjects with NEC Bell’s stage ≥II, significantly lower average rsSO_2_** (32.6% vs. 47.0%), **more time with rsSO_2_ < 30%** (48.8% vs. 6.7%) at one-fourth-volume feeds, **more time with SpO_2_-rsSO_2_ exceeding 50%** (33.3 vs. 0%) before feeds initiation compared to subjects with no or suspected NEC (all *p* = or <0.05)
**Abdominal US**	*Lazow* et al. *Pediatr Surg Int 2022* [81] *Neonates*	Multicenter retrospective review of 86 patients with NEC with CHD (n = 18, GA 37, BW 2.4 kg) vs. without CHD (n = 68, GA 27.6, BW 1 kg)	- **Less US findings of pneumatosis and of decreased mural flow in CHD patients** compared to non-CHD patients (33.3% vs. 72.1% with *p* = 0.005, 0% vs. 20.6% with *p* = 0.035) - 3.9 fold more discordant studies with pneumatosis on AXR but not on AUS in CHD patients (33.3% vs. 8.8%, *p* = 0.016) - No difference in US findings of portal venous gas, echogenic or hyperemic bowel, pneumoperitoneum, and fluid collection
	*Abraham* et al. *Pediatr Cardiol 2012* [82] *Neonate*	Single case report of a 5-day-old term neonate with CAVC with Down syndrome (term neonate, BW 3 kg)	Stream of spontaneous air contrast tracked from the hepatic veins/portal veins and entering the heart via the inferior vena cava, proven to be present because of pneumatosis at diagnosis of NEC
	*Müller* et al. *Eur J Pediatr 2014* [83] *Neonate/Infant*	Single case report of a 5-week-old boy with PA and IVS (GA 39, BW 3.5 kg)	Free air in right atrium as suspicious sign of NEC at the time of diagnosis
	*Cheong* et al. *Cureus 14(3):e22970* [84] *Neonate*	Single case report of a 6-day-old neonate with postnatal diagnosis of complex CHD (IAA type B, VSD) developing fulminant NEC after obstructive shock with end-organ damage (term neonate, BW 2.7 kg)	Systemic air embolism (widespread intra-vascular microbubbles entering from the portal veins, liver parenchyma, hepatic veins, right atrium, across the ventricular septal defect, and eventually into the systemic circulation) in the presence of NEC (day 20 of life)

**Abbreviations:** AUS = abdominal ultrasound; AXR = abdominal X-ray; BV = biventricular, BW = birth weight; CAVC = complete atrio-ventricular canal, CHD = congenital heart disease, Dao = descending aorta; DD = ductus-dependent; GA = gestational age; GLS = global longitudinal strain; IVS = intact ventricular septum, MAPCAs = multiple aortopulmonary collateral vessels; MRI = magnetic resonance imaging; MV = mean velocity, MV = mechanical ventilation; NIRS = near-infrared spectroscopy; PA = pulmonary atresia; PI = pulsatility index; PSV = peak systolic velocity; rsSO_2_ = regional splanchnic oxygen saturation, Se = sensitivity; Sp = specificity; SV = single ventricle.

## Data Availability

No new data were created or analyzed in this study.

## Supplementary Materials

The following supporting information can be downloaded at: www.mdpi.com/article/10.3390/children11111343/s1.
