# Supplementary material for: Noninvasive Tools to Predict Necrotizing Enterocolitis in Infants with Congenital Heart Diseases: A Narrative Review
Source: Children (Basel). 2024 Oct 31;11(11):1343. doi: 10.3390/children11111343 (PMC11592962; doi:10.3390/children11111343)
Supplement: Supplementary file 1 [file children-11-01343-s001.zip › children-3283786-supplementary.pdf]

Supplementary Material

Non-invasive tools to predict necrotizing enterocolitis in infants with congenital heart diseases: a narrative review

Laura Moschino, Silvia Guiducci Miriam Duci, Leonardo Meggiolaro, Daniel Nardo, Luca Bonadies, Sabrina Salvadori, Giovanna Verlato, Eugenio Baraldi

Literature search strategy

PubMed
(("neonate"[All Fields] OR "neonates"[All Fields] OR "newborn"[All Fields] OR "newborns"[All Fields] OR "infant"[All Fields] OR "infants"[All Fields]) AND ("congenital heart disease"[All Fields] OR "heart disease"[All Fields] OR "CHD"[All Fields]) AND ("necrotizing enterocolitis"[All Fields] OR "NEC"[All Fields]))

Medline Ovid
((neonate.af. OR neonates.af. OR newborn.af. OR newborns.af. OR infant.af. OR infants.af.) AND (congenital heart disease.af. OR heart disease.af. OR CHD.af.) AND (necrotizing enterocolitis.af. OR NEC.af.))

Scopus
TITLE-ABS-KEY (((neonate OR neonates OR newborn OR newborns OR infant OR infants) AND ("congenital heart disease" OR "heart disease" OR chd) AND ("necrotizing enterocolitis" OR nec)))

Results of the literature search up to August 2023
Table

Database	References	References after title and abstract screening
**PubMed**	193	11
**Ovid MEDLINE**	154	10
**Scopus**	568	5
**Total articles**	915	18 (after duplicates' removal and adjunct of articles from manual search)
